# Changes in Gene Expression Associated with Matrix Turnover, Chondrocyte Proliferation and Hypertrophy in the Bovine Growth Plate

**Published:** 2014

**Authors:** E. V. Tchetina, F. Mwale, A. R. Poole

**Affiliations:** Clinical Immunology Department, Research Institute of Rheumatology, Russian Academy of Sciences, Moscow, 115522, Russia; Orthopaedics Research Laboratory, Jewish General Hospital, Lady Davis Institute for Medical Research, Division of Orthopedic Surgery, McGill University, Montreal, Quebec, H3T 1E2, Canada; Department of Surgery, McGill University, McGill University, Montreal, Quebec, H3A 0G4, Canada

**Keywords:** growth plate, gene expression, proteinases, chondrocyte differentiation

## Abstract

The aim of the study is to investigate the interrelationships between the expression of genes for structural extracellular matrix molecules, proteinases and their inhibitors in the bovine fetal growth plate. This was analyzed by RT-PCR in microsections of the proximal tibial growth plate of bovine fetuses in relationship to expression of genes associated with chondrocyte proliferation, apoptosis, and matrix vascularization. In the resting zone the genes for extracellular matrix molecule synthesis were expressed. Extracellular matrix degrading enzymes and their inhibitors were also expressed here. Onset of proliferation involved cyclic upregulation of cell division-associated activity and reduced expression of extracellular matrix molecules. Later in the proliferative zone we noted transient expression of proteinases and their inhibitors, extracellular matrix molecules, as well as activity associated with vascularization and apoptosis. With the onset of hypertrophy expression of proteinases and their inhibitors, extracellular matrix molecules, as well as activity associated with vascularization and apoptosis were significantly upregulated. Terminal differentiation was characterized by high expression of proteinases and their inhibitors, extracellular matrix molecules, as well as activity associated with apoptosis. This study reveals the complex interrelationships of gene expression in the physis that accompany matrix assembly, resorption, chondrocyte proliferation, hypertrophy, vascularization and cell death while principal zones of the growth plate are characterized by a distinct signature profile of gene expression.

## INTRODUCTION

Endochondral ossification is a process involving chondrogenesis, chondrocyte hypertrophy, matrix mineralization, and vascularization followed by bone formation [1]. It begins during long bone formation in the embryo. After birth until adulthood, growth of the long bone is centered in the cartilagenous growth plates, leading to an increase in bone length and epiphyseal growth. It is also an essential component of fracture repair.

In the growth plates distinct zones can be observed. Cells of the resting zone chondrocytes produce large amounts of extracellular matrix (ECM). In contrast cells of the proliferative zone divide to give rise to columns of flattened cells that also secrete an ECM. At this time they express cell cycle-related genes such as cyclins. In the zone of maturation the cells round up and begin to enlarge into hypertrophic chondrocytes. The upper hypertrophic zone is characterized by cells that have enlarged 5- to 10-fold by a reduction in matrix volume per total tissue volume and which synthesize type X collagen [2-4]. In the lower hypertrophic zone calcification of the extracellular matrix occurs mainly in the longitudinal septa. The mineralization process, in combination with low oxygen tension, attracts blood vessels from the underlying primary spongiosum. Subsequently, the mineralized chondrocytes undergo apoptotic cell death [5].

The ECM of chondrocytes is a complex structure although 3 structural entities can be distinguished [6]. One of them is the complex of aggrecan molecules bound to hyaluronan and assembled into large aggregates. It is responsible for the cartilage compressive stiffness creating a highly hydrated matrix the expansion of which is constrained by a network of collagen fibrils composed of type II collagen, as well as a filamentous network of type VI collagen. Type II collagen fibrils contain a number of molecules at their surface, such as type IX collagen, decorin and fibromodulin. The key role of this network is to provide the tensile properties of this tissue. The non-fibrilar filaments of type VI collagen are involved both in cell-matrix and matrix- matrix interactions [6].

Changes in composition of the ECM occur as chondrocytes divide and mature. Metalloproteinases (MMPs) are generally considered to play a principal role in the cleavage of matrix macromolecules including type II collagen and aggrecan [3]. Only collagenases such as MMP-13, MMP-14 and cathepsin K, are capable of cleaving the triple helix of type II collagen [6]. This results in the unwinding (denaturation) of the triple helical domain which becomes susceptible to secondary cleavage by collagenases and other metalloproteinases such as stromelysin-1 (MMP-3) and gelatinases A and B (MMP-2 and MMP-9, respectively) [7]. MMP-13 is involved in the resorption of type II collagen that occurs during chondrocyte hyperthropy [8, 9]. Proteoglycan aggrecan can be cleaved by MMPs and by aggrecanases -1 and –2, ADAMTS-4 and ADAMTS-5, respectively [10]. In contrast the mechanism of type VI collagen degradation remains unclear. It is resistant to several extracellular matrix metalloproteinases in vitro including collagenases [11]. In cartilage MMP-2 or membrane-bound MMPs may be involved in its cleavage [12].

The activity of MMPs is further regulated by a family of specific inhibitors - tissue inhibitors of metalloproteinases, namely TIMPs –1, -2, -3 and -4 [13]. TIMP-1 and -2 inhibit the activity of all MMPs, whereas TIMP- 3 only inhibits MMP-1, -2, -3, -9 and -13 [14]. Besides inhibiting MMPs, TIMPs also appear to perform other functions. TIMP-1 and -2 exhibit growth factor activity [15] and TIMP-3 is an active mitogen [16].

The complex coordinated regulation of chondrocyte maturation in the growth plate is exerted both by the systemic hormones and chondrocyte autocrine growth factors [5]. In our previous studies of the bovine growth plate we have shown two peaks of gene expression [17]. An increase in gene expression in the early proliferative zone was associated with the upregulation of the regulatory growth factors *FGF-2 *and *PTHrP*. In contrast the second more pronounced peak of gene expression in the early hypertrophic zone was accompanied by the increase in *Cbfa1, TGFβ1 *and *Indian hedgehog (Ihh) *expression. In the present study we extend the previous investigations to explore the relationships of gene expression patterns of matrix proteins to other proteinases and their inhibitors to the cellular and extracellular changes that occur in the physis of the bovine growth plate. These observations help provide more insight into the complex interrelationships of the expression of these molecules during this critical stage in endochondral ossification.

## EXPERIMENTAL

**Tissue Preparation**

Bovine fetuses obtained from a local abbatoir immediately after the slaughter of pregnant cows, were transported to the laboratory. Fetal age was determined by measurement of tibial length [18]. Fetuses ranged from 190 to 210 days old. Tissue preparation was essentially as described [2, 8]. Only blocks of growth plate with a flat fracture surface were used. Tissue blocks were trimmed to provide cross-sectional areas of approximately 25 mm2. One hundred micrometer thick transverse sections were cut parallel to the fracture face (using a Vibratome; Ted Pella, Inc., California, USA), starting at the fracture face and extending through the hypertrophic zone into the upper proliferative zone of fetal bovine growth plate. They represented tissue labeled as A, B, C, and so on, from the fracture face. Their locations have been previously characterized [2, 8]. A series of sections of four growth plates was pooled (A with A and B with B, etc.) to permit collection of a sufficient amount of tissue for the analyses. Wet weights were determined immediately after sectioning: the weights ranged from 10 to 15 mg, depending on the sample. The weights of samples A and B were lower due to some irregularity of the fracture face.

**Total RNA Isolation and Reverse Transcriptase Polymerase Chain Reaction (RT-PCR)**

**Fig. 1:**
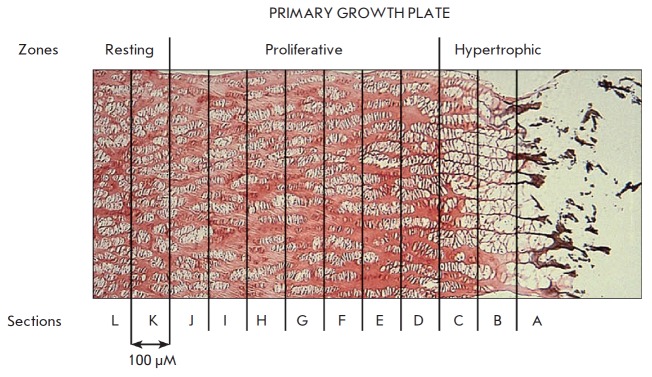
Representation of the organization of the primary growth plate showing sampling sites extending from the hypertrophic zone through the proliferative zone into the resting zone

Total RNA was isolated by a modification of the method of Chomczinski and Sacchi, which was described previously [9]. The RT-reaction was performed using total RNA isolated from the cartilage in a total volume of 20 m1 using SuperScript TMII H-Reverse Transcriptase (as recommended by Invitrogen, Canada, Inc.).

**Fig. 2:**
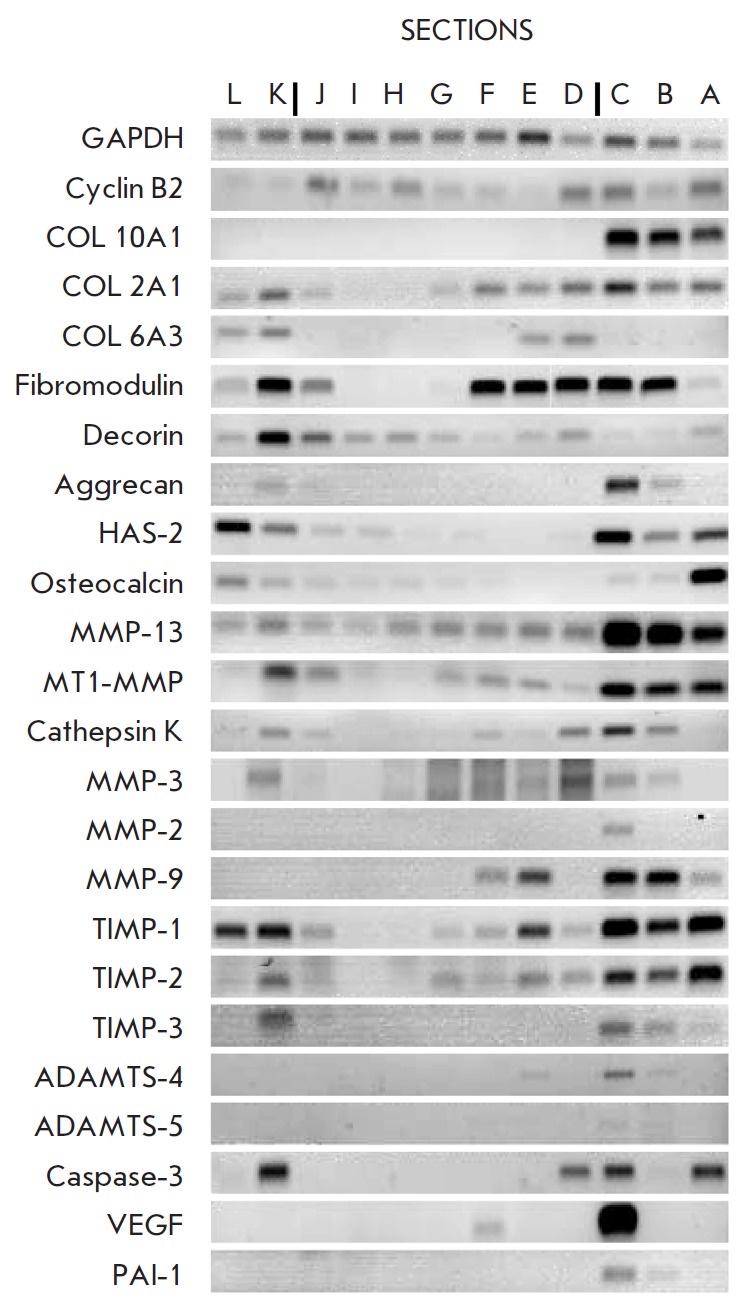
A representative RT-PCR analyses of gene expression in the zones of the growth plate

**Fig. 3:**
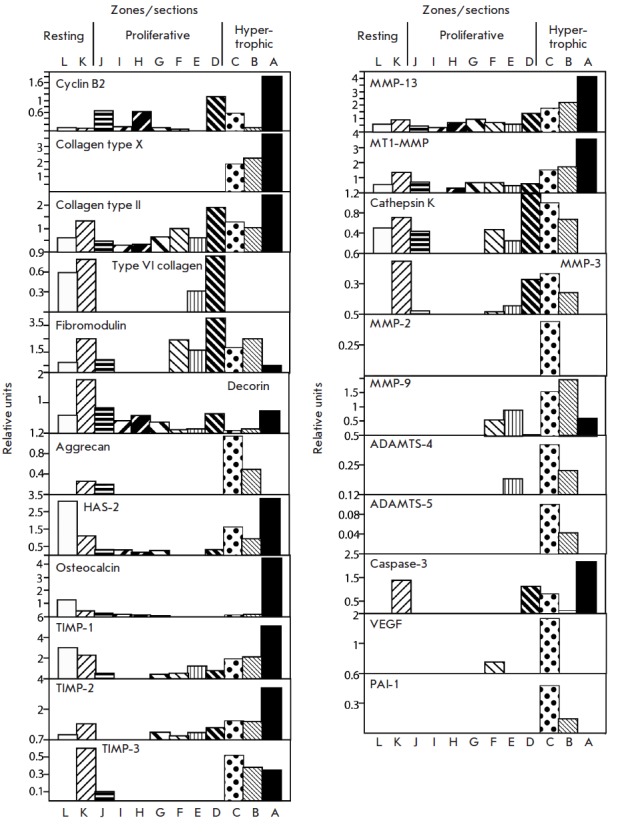
Relative RT-PCR analysis of gene expression shown in Fig. 2 in relationship to GAPDH expression determined using NIH1.60 software

Oligo sequences used for PCR are shown in *Table 1*. PCR was performed in a total volume of 25 μ1 containing: 10 mM Tris-HCl, pH 8.3, 1.5 mM MgCl2 , 0.4 mM each of dATP, dGTP, dCTP, dTTP, 0.8 mM of each primer, 1m1 of RT mixture and 2.5 units of AmpliTaq DNA polymerase (Perkin Elmer). The 30 cycles of PCR included denaturation (95°C, 1 min), annealing (50°C, 1 min) and extension (72°C, 5 min). After agarose (1.6%) gel electrophoresis, PCR products were visualized by ethidium bromide staining. GAPDH was used as reference for gel loading. The band intensities were determined to be below saturation by dilution analyses. Each analysis was performed at least 3 times at different dilutions of each sample of the original cDNA. The result of the single dilution for all the samples in a given set which showed most clearly differences in expression (e.g.COL2A1) is presented in *Fig. 2* and *3*. Results were analyzed using NIH 1.60 software to determine the pixel intensity for each band and autobackground subtraction was used to control for background signal (*Fig. 3*). These results were reproducible for growth plates from three different fetuses.

The isolated clones of each amplified cDNA fragment were sequenced (Sheldon Center, McGill University) to verify the identity of each cDNA product. To confirm the lack of chromosomal DNA contamination of RN A samples, PCR was also performed with RN A aliquots. To avoid variation in efficiency between experiments, all sections were simultaneously subjected to reverse transcription and all samples of cDNA were simultaneously amplified in PCR.

**Table 1:** Oligo sequences used for PCR

Genes	Forward primer	Reverse primer
Collagenase 3 (MMP-13)	GATAAAGACTATCCGAGAC	GAGTAACCGTATTGTTCG
Membrane type 1-MMP (MMP-14)	GCATCCAGCAACTTTATG	CATCTGTGACGGGAACTTTG
Stromelysin-1 (MMP-3)	TGCGTGGCAGTTTGCTCAGCC	GAGGTGACTCCACTCACATTC
Gelatinase A (MMP-2)	GCTACACACCTGATCTG	GACGGCAAGTATTGTTCTG
Gelatinase B (MMP-9)	GCAGAGGAATACCTGTAC	CACAACATCACCTACTG
Tissue inhibitor of metalloproteinase-1 (TIMP-1)	GAAAACTGCAGGATGGAC	CACCAAGACCTACACTGTTG
Tissue inhibitor of metalloproteinase-2 (TIMP-2)	GGATATAGAGTTTATCTACAC	CATGATCCCGTGCTACATCTC
Tissue inhibitor of metalloproteinase-3 (TIMP-3)	CTTAGGCTGGAGGTCAACAAG	CAAGAACGAGTGTCTGGAC
Osteocalcin (bone Gla protein)	CTTTGTGTCCAAGCAGGA	CTATCGGCGCTTCTAC
Procollagen type II (COL2A1)	GAACCCAGAACAACACAATCC	GTTCGGACTTTTCTCCCCTCT
Procollagen type X (COL10A1)	CTGAGCGATACCAAACACC	GTAAAGGTGTATCACTGAGAGG
Cyclin B2	GTTGACTATGACATGGTG	GTTCGTGCACTTTGTCTTG
Procollagen type VI (COL6A3)	CATGCTTTGATTTACACTCG	CACTGCTGGTGTTTATGTG
Fibromodulin	CAAGGCAATAGGATCAATG	GTTTGGCTTATGGAAGGTC
Decorin	TGAGTTTCAACAGCATCTCTGC	GTGAGCCTTTTCAGCAACC
Aggrecan	TGAGGAGGGCTGGAACAAGTACC	TGTTCCCTGCAATTACCACCTCC
Hyaluronan synthase 2 (HAS-2)	CTCATCAATAAGTGTGGCAG	CCTATATACCTCACTAGCC
Cathepsin K	GTGTGTCTGAGAATGATGGCTG	CAGCAAAGGTGTGTATTATG
Aggrecanase-1 (ADAMTS-4)	ACCACTTTGACACAGCCATTC	TGCTCTCGGACCTGTGGGGGT
Aggrecanase-2 (ADAMTS-5)	TGTGCTGTGATTGAAGACGAT	CCATCTACCGCTCCTGCAC
Caspase 3	CTGGTACAGATGTCGATGCAG	CATTGAGACAGACAGTGGTG
Vascular endothelial growth factor (VEGF)	GTTCATGGATGTCTATCAG	GCACAACAAATGTGAATGCAG
Plasminogen activator inhibitor (PAI-1)	GATCCAAGAGGCAATGCAATTC	GATCAGCGACTTACTTGGTG
Glyceraldehyde 3-phosphate dehydrogenase (GAPDH)	GCTCTCCAGAACATCATCCCTGCC	AGCTCATTTCCTGGTATGACAACG

## RESULTS AND DISCUSSION

Sequential transverse sections of the bovine tibial primary proximal growth plate (*Fig. 1*), which represent the hypertrophic (A-C), proliferative (D-J) and resting (K-L) zones [17], were generated. Using RT-PCR analyses of sequential transverse sections of the growth plate cartilage the expression of markers of chondrocyte proliferation and terminal differentiation has already been determined [17]. Here we present our analyses of gene expression of ECM proteins, HA synthase-2, and proteinases in the bovine fetal growth plate in the course of chondrocyte differentiation. We repeated these analyses several times on different fetuses. The data that is shown is representative of our repeated analyses. The data describing the expression of *GAPDH, cyclin B2, COL2A1, COL10A1, osteocalcin, MMP-13 *and *MMP-9 *is reproduced from our previous study [17] for reference.

In our RT-PCR analyses (*Fig.2*) and its schematic presentation in relationship to *GAPDH *expression (*Fig.3*) the onset of proliferation was defined at section J where the upregulation of the *cyclin B2 *expression is observed. The onset of terminal differentiation was considered as sample C, where the expression of *COL10A1 *the marker of hypertrophic chondrocytes is first detected.

**The resting zone**

The resting zone (sections L and K) is characterized by the expression of extracellular matrix genes, namely* COL2A1, COL6A3*, *fibromodulin *and *decorin *being highest in section K. *HAS-2 *expression is more pronounced (in section L) than that of *aggrecan *in section K. *Osteocalcin *expression is also detected in section L. Of the proteinases tested *cathepsin K, MMP-14, MMP-13 *(weakly) and *MMP-3 *were all expressed in this zone. In contrast the expressions of the MMP inhibitors *TIMP-1 *(strongly) and *TIMP-2 *(weakly) and* TIMP-3 *(strongly but only in section K) were detected. In contrast there was no expression of the gelatinases* MMP-2, MMP–9 *nor of the aggrecanases *ADAMTS-4* and *–5 *in sections L and K. *Caspase 3 *was expressed only in section K where *MMP-3 *and *TIMP-3 *expression was strong.

**Proliferative zone**

The upregulation of *cyclin B2 *in section J indicates the beginning of chondrocyte proliferation in the growth plate. This is associated with the downregulation of expression of all matrix proteins tested, namely *COL2A1, aggrecan, HAS-2, fibromodulin *and *decorin, osteocalcin* as well as *TIMPs *and proteinases previously upregulated. No expression of *COL6A3 *and *caspase 3 *was detected in this section and expression did not reappear until the lower proliferative zone in section E and D respectively. The expressions of *aggrecan, HAS-2, osteocalcin, TIMPs, MMP-13 *and *MMP-14, ADAMTS-4 *and *–5 *were absent or markedly reduced in the central proliferative zone.

In section I *cyclin B2 *expression was downregulated although its expression level was up again in section H dropping until section D when it rose again. Expression of *COL2A1 *was maintained until section D when it rose with that of *COL6A3, fibromodulin, decorin *and* cathepsin K, MMP-13, MMP-3 *and *caspase 3*. Chondrocytes in section E also expressed *COL6A3, fibromodulin, ADAMTS-4 *and *MMP-9*, which also rise in section F. Expression of *TIMPs-1 *and *–2 *started to rise again, where *MMP-13 *and *MMP-14 *were weakly coexpressed with *MMP-3 *in this region.

*Caspase 3 *expression in section D immediately preceded that of the hypertrophic chondrocytes phenotype identified by expression of *COL10A1 *in section C. This is where *cathepsin K *expression reached a peak with *MMP-3 *and *fibromodulin, COL2A1 *and *COL6A3*. Here *cyclin B2 *was again elevated.

**Hypertrophic zone**

The onset of hypertrophy in section C was accompanied by the strongest but transient expression of *VEGF* as well as *aggrecan *and *HAS-2*. *COL2A1 *expression was maintained but that of *COL6A3 *was not detected in this zone. In section C *MMP-13 *continued to rise with* MMP-14, MMP-3, MMP-2 *(latter only expressed here) and *MMP-9. ADAMTS-4 *and *ADAMTS-5 *were strongly expressed with *cathepsin K*. The inhibitors *TIMP-3* and *PAI-1 *were markedly increased here.

In section B the proteinases continued to be expressed (except *MMP-2 *which was only expressed in section C) but less so in the case of the aggrecanases. The inhibitors were also expressed but *PAI-1 *expression was reduced.

The late hypertrophic zone of bovine growth plate is represented by section A. The gene expression analysis of this section revealed the highest expression of *cyclin B2, caspase 3, COL10A1, COL2A1*, *HAS-2 *and *osteocalcin*. Collagenases *MMP-13 *and *MMP-14 *were also maximally expressed but *MMP-2, MMP-3, cathepsin K *and *ADAMTS-4 *and *-5 *were not expressed. *MMP-9* was weakly expressed. *Aggrecan *and *fibromodulin *expression was absent and weak, respectively. *TIMPs –1* and *–2 *were further elevated, *TIMP-3 *unchanged and* PAI-1 *was absent.

The microanalytical methodology, involving RTPCR analyses of sequential transverse sections of the primary proximal tibial growth plate, used in this and our previous study [17] permits expression analyses of the interrelationships of genes that have usually been studied individually concerning their involvement in chondrocyte differentiation, matrix assembly and remodeling in the growth plate.

From our present studies, combined with our earlier analyses, we can observe that each of the principal zones of the growth plate is characterized by a distinct signature profile of gene expression. Thus the resting zone (sections L and K) is characterized by the expression of matrix molecules that include the collagen fibrillar network of *COL2A1 *and the associated proteoglycans* fibromodulin *and *decorin*, the filamentous collagenous network of *COL6A3*, the aggrecan network with *HAS-2 *representing the synthesis of hyaluronan, a key component of aggregating proteoglycans. There is even a low level of expression of *osteocalcin* better known as a protein expressed by osteoblasts and terminally hypertrophic chondrocytes [19]. This matrix gene expression is associated with expression of *caspase 3 *for reasons that are unclear. Moreover, a low expression level of the proteinases *MMP-3, –13, -14 *is also seen, accompanied by expression of all three* TIMPs*. Although MMPs are regulated both at gene expression and protein level, the correspondent local increase of collagenase dependent collagen cleavage activity at (next to) this area of the growth plate has been also observed by us earlier [8]. This expression of matrix degradation genes is associated with expression of *caspase 3*, indicating the cell apoptosis which accompanies chondrocyte proliferation in animal growth plate [20]. However at this time matrix assembly dominates but is accompanied by limited matrix remodelling as was suggested by our previous direct analyses of matrix collagen and proteoglycan in this growth plate [8]. The increased expression of *cathepsin K *in this zone raises questions as to whether this is related to either extracellular and /or intracellular activity of this proteinase. At this stage it is worthy of note that* ADAMTS-4 *and *–5 *are not expressed until the hypertrophic zone and that evidence for their involvement in aggrecan degradation is not seen until hypertrophy is observed. Upregulation of matrix remodeling genes in the area adjacent to the beginning of chondrocyte proliferative activity is associated with the strong upregulation of proliferative zone related growth factors, namely *FGF-2, TGFβ2 *and *PTHrP *[17] indicating their involvement in the regulation of matrix turnover.

The upper proliferating growth plate chondrocytes, delineated by the increased expression of *cyclin B2*, which is first observed in section J and then H. These early proliferative chondrocytes did not show any significant changes in relative expression of genes involved in matrix remodeling.

The downregulation of *cyclin B2 *expression in section E, preceding hypertrophy and is associated with another expression maximum of matrix remodeling. In contrast to resting zone, at this time the upregulation of matrix proteins *COL2A1, COL6A3 *and *fibromodulin* is not accompanied by significant increase in *decorin, aggrecan *and *HAS-2 *expression. However, as in the resting zone, expression of matrix degrading genes* MMPs *and *ADAMTS-4 *and their inhibitiors *TIMPs *was detected. *Fibromodulin *has been shown to be strongly expressed only in the proliferative zone in the rat and mouse growth plates [6, 21]. In contrast previous studies using sequential transverse sections (200-400mm) of bovine growth plate revealed the presence of *fibromodulin* message in all the zones except the lower hypertrophic [22]. Our analyses reveal a 300mm region of the proliferating zone lacking significant expression of this protein. This study has also revealed that the distribution of *fibromodulin *expression in bovine growth plate is similar to that of *type II collagen *as was seen in mouse growth plate [23].

The gene expression of another collagen binding proteoglycan *decorin *progressively decreases in the proliferative zone confirming earlier data [21, 22]. This may be related to the ability of decorin to inhibit bone mineralization [23] which we know starts in section H in proliferating bovine growth plate chondrocytes [8].* Decorin *expression is clearly greatest in the resting zone and decreases prior to hypertrophy.

In general the gene expression pattern in section D immediately preceding the hypertrophic zone is similar to that immediately preceding the onset of proliferation (section K) characterized by the expression of *COL2A1, COL6A3, fibromodulin, decorin, MMP-13, MMP- 14, cathepsin K, MMP-3, TIMPs –1, –2 *and *caspase 3*. However, at this time there is no *aggrecan*, little *HAS-2* expression and *TIMP-3 *expression is also lacking. In spite of the similarity in gene expression pattern in sections K and D including that of *caspase 3*, the further fate of both groups of growth plate chondrocytes is not the same. Instead of chondrocyte progression to proliferation it is now to hypertrophy and is accompanied by the expression of different regulatory growth factors: namely *PTHrP *and *FGF-2 *at the onset of proliferation, and *TGFβ1 *and *Ihh *in the hypertrophic zone [17].

Immediately prior to hypertrophy there are some clear-cut changes in expression. *COL6A3 *transiently peaks again as does *fibromodulin*. *Type II collagen *expression is also upregulated at this time. As we mentioned previously [17], *COL2A1 *expression was detected throughout the growth plate. But when PCR was performed using equally diluted samples, three peaks of *COL2A1 *expression were observed in samples K, D, and A. The highest level of *type II collagen *expression in the lower proliferative and upper hypertrophic zones was also observed by others [24]. *MMP-9 *is upregulated for the first time as is *ADAMTS-4*, although both transiently at this stage. *Cathepsin K *and *caspase 3 *both rise again. The upregulation of the expression of these two genes in the proliferative and early hypertrophic chondrocytes were also observed in rodent and human growth plates [20, 25]. Clearly these changes reflect the cessation of proliferation and the beginning of hypertrophy.

The onset of hypertrophy is characterized by the sudden expression of *COL10A1*. This another gene expression maximum is characterized by the upregulation of *COL2A1, fibromodulin, aggrecan *and *HAS-2* expression and downregulation of *COL6A3*. The active process of ECM remodeling involving type II collagen loss mediated by collagenase [8] is accompanied by the upregulation of all the collagenases, gelatinases (*MMP- 2 *and *MMP-9*), *MMP-3, TIMPs *and expression of the aggrecanases *ADAMTS-4 *and -*5*.

Growth plate vascularization is associated with the early transient hypertrophic upregulation of *VEGF* and persistent upregulation of *MMP-9 *expression as observed by others [26, 27]. *MMP-9 *expression clearly accompanies the expression of *VEGF *which is a chemoattractant and a mitogen for endothelial cells [28]. Active blood vessels ingrowth in the hypertrophic zone of the growth plate may account for upregulation of* cyclin B2 *expression also seen in section D, C and A.

The final maximum of gene expression in section A is associated with the strong upregulation of collagenases* MMP-13 *and *MMP-14*, the loss of expression of *cathepsin K, *and *ADAMTS-4 *and *-5 *and the maintenance or an increased in expression of the MMP inhibitors* TIMP-1, TIMP-2 and TIMP-3 *and is accompanied by an increase in the expression of *COL2A1, decorin *and* HAS-2*. No expression of *aggrecan *or *type VI collagen *is detected at that time but *osteocalcin *is again expressed. The downregulation of *fibromodulin *expression seen here in the late hypertrophic zone has previously been established [21, 22].

Overall, by using the enlarged bovine physis our study provides an original insight into the interrelationships of gene expression in chondrocyte proliferation and differentiation associated with extracellular matrix assembly, mineralization, and vascularization. Our approach is the first sequential presentation of various genes in one study that permits an analysis of individual gene expression changes associated both with respect to their alterations in the continuum of chondrocyte differentiation ending in cell death through the growth plate. It also allows for a comparison of the expression of various genes in each individual 100um zone of the bovine physis.

In this respect upregulation of a gene in a distinct zone of the growth plate indicates its involvement in the processes associated with exact phase of chondrocyte differentiation. In contrast, downregulation of a gene indicates that its function is less important in that zone of the growth plate. In view of this the previously observed biphasic character of *MMP-13 *expression in rodent growth plate [29] was supplemented by our original observation that that is not a case for *MMP-9* and -2, expressions of which were associated only with pre-hypertrophic and hypertrophic phases of chondrocyte differentiation. This further indicates the importance of collagenases MMP-13, MT1-MMP, MMP-3, and cathepsin K in extracellular matrix remodeling associated with further synthesis of chondrocyte-specific matrix supported by upregulation of extracellular matrix-related molecule expression here and in the following proliferative zone of the growth plate. In contrast, upregulation of *MMP-9*, *-2*, and both *aggrecanases* associated only with chondrocyte hypertrophy indicates their destructive activity in respect to chondrocyte- specific matrix. Moreover, the observed differences in matrix degrading molecule expression might be related also to differences in regulation of their expression as we previously reported [17] and differential growth factor profiles associated with early proliferative and hypertrophic zones in the bovine growth plate.

It is worth noting that early upregulation of genes involved in mineralization in the midst of proliferative zone in bovine growth plate observed in our previous studies [8] is also associated with upregulation of the genes related to extracellular matrix-related molecule expression, their inhibitors and vascularization markers: overt mineralization occurs later in the hypertrophic zone. This suggests that any alteration in chondrocyte metabolic activity is associated with specific extracellular matrix remodeling, which affects its properties and subsequent bone formation.

Therefore, our results indicating fluctuations in gene expression for extracellular matrix molecules, proteinases and their inhibitors in the bovine growth plate were expected. However, the exact profile of each gene pattern could not be predicted with accuracy prior to completion of this study.

## CONCLUSIONS

The data presented here further define the complex changes and interrelationships in gene expression in the physis of the growth plate that occur in the course of chondrocyte maturation associated with matrix assembly, remodeling, cell proliferation, differentiation, vascular invasion and cell death. This investigation draws attention to distinct phases of expression of matrix molecules, proteinases and their inhibitors and their relationships to the physiological events and regulatory molecules that are part of endochondral ossification.

## Abbreviations

ECM: extracellular matrix
MMP: metalloproteinase
TIMP: tissue inhibitor of metalloproteinases
HAS: hyaluronic acid synthase
COL: collagen
ADAMTS: A Disintegrin And Metalloproteinase with Thrombospondin Motifs
FGF: fibroblast growth factor
PTHrP: parathyroid hormone related peptide
Cbfa1: core-binding factor subunit alpha-1 (CBF-alpha-1)
TGFb1: transforming growth factor beta 1
Ihh: Indian hedgehog
VEGF: vascular endothelial growth factor
PAI-1: plasminogen activator inhibitor-1
GAPDH: glyceraldehyde 3-phosphate dehydrogenase
RNA: ribonucleic acid
RT-PCR: Reverse Transcriptase Polymerase Chain Reaction
cDNA: complementary DNA
